# Foot Involvement as the First Manifestation in Rheumatoid Arthritis Patients in Lahore

**DOI:** 10.7759/cureus.15347

**Published:** 2021-05-31

**Authors:** Sajid Ali Khan, Muhammad Ahmad Saeed, Sumaira Farman, Zarghoonah Sajid, Nighat Ahmad, Masood Alam

**Affiliations:** 1 Rheumatology, Fatima Memorial Hospital College of Medicine and Dentistry, Lahore, PAK; 2 Medicine, Mian Muhammad Shahbaz Sharif Hospital, Lahore, PAK; 3 Pulmonology, Chaudhary Pervaiz Elahi Institute of Cardiology, Multan, PAK

**Keywords:** foot involvement, rheumatoid arthritis, walking difficulty, ra factor, anti ccp antibodies

## Abstract

Objective

The objective of this study was to determine the involvement of the foot as the first manifestation in rheumatoid arthritis (RA).

Methods

This study was conducted on 100 patients who presented to the outpatient department of the Rheumatology Department at Fatima Memorial Hospital and College of Medicine and Dentistry in Lahore, Pakistan. The period of this cross-sectional study was three months - from October 2017 to December 2017. One hundred patients aged more than 16 years, who were diagnosed as cases of RA with foot involvement, and with a disease duration of less than two years (to minimize recall bias), were enrolled for the study.

Results

Out of the 100 patients, 20% were male and 80% were female. The mean age of the study population was 41.16 ± 12.343 years. Foot Involvement as the first manifestation was noted in 29 (29%) of the cases. The pattern of foot involvement shows a 59% forefoot involvement, 27% hindfoot involvement, and 14% midfoot involvement. Walking difficulty was most common in forefoot involvement (71.42% of cases), followed by hindfoot involvement (20% of cases), and least common in midfoot involvement (8.57% of cases) (p=0.0001).

Conclusion

Foot involvement as the first manifestation is quite common in RA and should not be ignored, especially in patients with large joint involvement sparing the hands.

## Introduction

Rheumatoid arthritis (RA) is the prototypical multisystem autoimmune disorder with a broad spectrum of joint presentations encompassing almost all joints and soft tissues. It has a 39% morbidity within 10 years of onset [1]. It is a disease that affects multiple joints, with the small joints of the wrist, hands, and feet being the most commonly affected joints [2]. RA is a disease of unknown etiology [3]. Its presentation and course are highly variable and range from minimal to fulminant [4].

Overall, in the population of the world, without any differences between developed and developing countries, rheumatoid arthritis affects 0.5-1.9% of people [5,6]. In Pakistan, the prevalence of RA is reported to be 0.142% in the southern regions [7] and 0.55% in the northern areas [8].

Persons with sensitivity to two HLA-DRB1 alleles are at more risk of developing RA than those who have a single-dose genotype [9]. A study conducted by Yahya A et al. in the Malaysian population reported that anti-citrullinated protein antibody (ACPA)-positive RA was observed in persons in the silica profession. Smokers exposed to silica dust are at a high risk of developing ACPA-positive RA [10].

Infections, as associated with other autoimmune disorders, have been extensively studied in RA. Although there is still no pathogen-derived antigen clearly linked to RA pathogenesis, the link between the human microbiome (particularly in the gut, oral cavity, and lungs) and the host's immune system is being intensively investigated. A change from a normal symbiotic relation to a “dysbiotic” microbiome is thought to be responsible for changes in both innate as well as the adaptive arm of immunity. This could be a factor in the development of RA [11].

The main types of arthritis cannot be differentiated on the basis of histology or the characteristics of cellular infiltrates. Therefore, a biopsy of the synovial tissue is rarely used as a diagnostic tool [12].

After the development of higher sensitivity criteria of 2010 American College of Rheumatology/European League Against Rheumatism (ACR/EULAR), a significant burden of the patients who were previously regarded as undifferentiated arthritis were categorized as rheumatoid arthritis [13]. The newly emerging criteria comprise the majority of clinically related parameters that were included in Leiden prediction rules. Their successful use helps discriminate between patients with high risk and those with low risk in terms of the radiological progression of the disease. In another trial called IMPROVED study, undifferentiated arthritis patients who did not fulfil the 2010 criteria were found to be less active, had lower tender joint count (TJC) and swollen joint count (SJC), and showed significantly lower proportions of ACPA and rheumatoid factor (RF) positive rates in comparison to RA (ACPA: 3% compared with 68%; RF: 4% versus 69%), as well as better prognostic features in terms of radiographic progression [14].

The strategy of treatment is always based on the "treat to target" approach. In this approach, one to three months of follow-up of the patient is required with proper treatment until the goal is achieved. Ten important recommendations were developed by the international task force of RA experts to educate patients and to guide practitioners in planning for better outcomes [15].

The prevalence of bacterial infection is very high in patients with RA due to the use of immunosuppressive (glucocorticoids) disease-modifying antirheumatic drugs (DMARDs) and biological agents. Through study results, we know that anti-tumour necrosis factor (TNF) therapy can double the risk of a serious infection [16].

Patients with RA are at risk of malignancy, like lymphoma in many patient subsets, especially with seropositive patients and those with longstanding disease. These patients have been advised that they are at risk of the severity and inflammatory activity of the disease. The anti-TNF drugs do not enhance the rate of malignancy, except in skin cancers, including melanoma and non-melanoma [17]. 

Approximately 13% of patients have foot involvement as the first sign of RA, and 90% of patients will have foot manifestations at some point during the course of the disease [18]. Foot involvement has been found to be the most common reason for disability in patients with RA, with the forefoot being the most common area [19]. According to a study conducted in 2012, at the site of the foot, the symptoms are mostly in the ankle (36%) and forefoot (30%), followed by the hindfoot (17%) and midfoot (7%) [20]. The deformities caused by foot involvement in RA include hallux valgus (HV), dorsal dislocation of the metatarsophalangeal (MTP) joints, and hammertoe deformity of the lesser toes. Changes to the forefoot that are found commonly in these patients include bunions, claw toes, “cock-up” deformity of the small toe, and pain affecting the ball of the foot. In some cases, the heel may also become painful. Despite the foot being a common cause of pain and disability, it is often overlooked in the clinical assessment of RA [16]. Foot involvement causes walking disability in three-quarters of the cases, which is four times as often as the hip or knee joint [18,19].

The new RA classification criteria were set as part of a joint ACR/EULAR effort in 2010 [20]. In these criteria, the small joints are given the maximum weightage, which is why foot involvement (having 33 small joints) as the first manifestation in RA should not be ignored. The purpose of this study is to enhance the sensitivity of the ACR criteria in making early diagnosis of RA in patients with the involvement of predominately large joints, sparing the hands but having foot involvement.

## Materials and methods

This was a prospective, cross-sectional analysis of 100 outpatients in the Department of Rheumatology, Fatima Memorial Hospital (FMH), which is a tertiary care hospital in Lahore city of Pakistan. All patients fulfilling the ACR/EULAR 2010 diagnostic criteria with foot involvement and having a disease duration of less than two years were enrolled in the study over a period of three months. All patients with degenerative diseases and anatomical abnormalities were excluded. The study was approved by the institutional review board of FMH. Informed consent was obtained from all subjects according to the 2008 Declaration of Helsinki.

The detailed history and clinical examination of the patients were recorded. Demographic data, such as age, gender, and area of residence, were noted on a form. The patients were asked to point out the first involved area of the body due to the disease. Every patient was asked to point out the area of foot involvement on homunculus divided into forefoot, midfoot, and hindfoot. The patients were asked if they had any difficulty walking. The status of RA factor and anti-cyclic citrullinated peptide (CCP) antibodies was recorded from their medical records.

All data was entered and analyzed in SPSS Statistics version 21 (IBM Corp., Armonk, USA). The quantitative variables were presented as mean and standard deviation. Frequencies and percentages were analyzed for the qualitative variables. A p-value of ≤0.05 was considered significant.

## Results

One hundred patients were enrolled in the study; n=20 (20%) were male and n=80 (80%) were female. The mean age of the study population was 41.16±12.34 years. Foot Involvement as the first manifestation was noted in n=29 (29%) cases. RA factor was positive in n=58 (58%) cases. Anti-CCP antibodies were positive in n=63 (63%) cases. The pattern of foot involvement showed n=59 (59%) cases of forefoot involvement, n=27 (27%) cases of hindfoot involvement, and n=14 (14%) cases of midfoot involvement (Table 1). Walking difficulty was most common in forefoot involvement (71.42% of cases), followed by hindfoot involvement (20% of cases), and the least common in midfoot involvement (8.57% of cases) (p=0.0001) (Table 2). Walking difficulty was significantly associated with the pattern of foot involvement, with the forefoot being the most commonly involved in walking difficulty (p=0.019 and 0.0001, respectively) (Tables 2, 3).

**Table 1:** Demographic characteristics of the patients RA: rheumatoid arthritis; CCP: cyclic citrullinated peptides

Variable	Frequency	Percentage
Gender		
Male	20	20.0%
Female	80	80.0%
Foot involvement as the first manifestation	29	29.0%
RA factor	58	58.0%
Anti-CCP antibodies	63	63.0%
Pattern of foot involvement in RA		
Forefoot involvement	59	59.0%
Hindfoot involvement	27	27%
Involvement of midfoot	14	14%

**Table 2:** Association between the pattern of foot involvement and walking difficulty

Pattern of Foot Involvement	Walking Difficulty		Total
	Yes	No	
Forefoot	50	9	59
Midfoot	6	8	14
Hindfoot	14	13	27
Total	70	30	100
Chi-square	15.225		
P-value	0.0001		

**Table 3:** Stratification of foot involvement as the first manifestation with respect to walking difficulty

Foot Involvement as First Manifestation	Walking Difficulty		Total
	Yes	No	
Yes	25	4	29
No	45	26	71
Total	70	30	100
Chi-square	5.109		
P-value	0.019		

## Discussion

There are no prior studies on foot involvement in RA in Pakistan. Although RA has been extensively studied, the foot has remained an understudied manifestation of rheumatoid arthritis, resulting in a higher proportion of missed diagnosis, especially in patients with large joint involvement sparing the hands.

This was a small-scale study to determine foot involvement in rheumatoid arthritis as the first manifestation. It showed that the foot is involved as the first manifestation in 29% of cases, which makes it a significant diagnostic consideration. The forefoot is the most commonly involved part of the foot and is also the most common cause of walking difficulty. Foot involvement is independent of the seropositive or seronegative status of the patient.

In one study by Grondal et al., the forefoot was involved in 45% of the patients, and the hindfoot/ankle was involved in 17% [21]. This supports our study which shows a 59% involvement of the forefoot. Difficulty in walking due to the feet was reported by 71% of the patients, which is almost the same as our study. For 41% of the patients, the foot was the most important part of the lower extremity that was causing reduced walking capacity - this is also similar to our study.

In a study by Keenan et al., it was demonstrated that the midfoot and ankle are the least commonly involved joints of the foot in RA [22]. This result also supports the results of our study.

Concerning RA, forefoot joint damage was significantly correlated with forefoot pressure [23], and RA patients showed lower medial and higher lateral forefoot peak pressures compared to healthy controls [24]. This explains the increased involvement of the forefoot and the higher incidence of walking difficulty with forefoot involvement that was observed in our study.

In a study by Michelson J et al., 93 out of 99 patients had complaints related to the foot or ankle at some time since their diagnosis of RA. Ankle problems were paramount in 42%, forefoot difficulties in 28% of patients, and 14% of the patients had equal ankle and forefoot problems [25]. These findings are in contrast to our observations, in which the forefoot is the most commonly involved part. Michelson et al. also concluded that their findings were opposite to other studies which indicate a more common involvement of forefoot. The reason for this discrepancy has not been discussed in the study.

In one study conducted in Singapore, Carter K et al. studied the prevalence of foot problems in people with inflammatory arthritis. They found that the foot is involved in 45% of cases in people with RA. However, no comment was made on the pattern of foot involvement or the walking difficulty caused by it [26]**.**

No local data is available on this subject in Pakistan or its neighbouring countries.

This is a small-scale study and needs further validation on a larger scale. However, it gives us a good insight into the pattern of foot involvement in rheumatoid arthritis on which future studies can be based.

## Conclusions

In this study, foot involvement as the first manifestation was seen in about one-third of the patients. This study suggests that clinicians taking care of patients with early inflammatory arthritis should perform a focused examination of the feet to look for synovitis involving the small joints of the feet. This may increase the sensitivity of the new ACR/EULAR criteria, especially in the patients in whom the small joints of hands are spared at the outset of the disease.
